# Treatment of Iatrogenic Aortocoronary Arteriovenous Fistula with Coronary Covered Stent

**DOI:** 10.1155/2016/9126817

**Published:** 2016-03-27

**Authors:** Ender Ornek, Harun Kundi, Emrullah Kiziltunc, Mustafa Cetin

**Affiliations:** Department of Cardiology, Ankara Numune Education and Research Hospital, Turkey

## Abstract

An 83-year-old man, who underwent coronary artery bypass operation of left internal mammary artery (LIMA) to left anterior descending (LAD) artery, with sequential saphenous vein to the first and second obtuse marginal (OM) branches of circumflex artery 5 years ago and coronary artery stent implantation to right coronary artery 2 months ago, was admitted to the hospital with syncope and chest pain. Aortosaphenous graft selective angiography revealed that first sequential side to side ligation was inadvertently anastomosed to left posterolateral coronary vein with resultant flow into the coronary sinus and distal end to side sequential anastomosis to OM 2 coronary artery which was filling very weakly. In order to close this iatrogenic coronary arteriovenous fistula and to supply saphenous vein flow to OM artery, we decided to implant a graft covered stent into the saphenous vein at the same session.

## 1. Introduction

Iatrogenic aortocoronary arteriovenous fistula resulting from placement of an arterial graft to a cardiac vein is a very rare complication of coronary artery bypass graft operation [1, 2]. Most patients present postoperatively with angina as a result of residual ischemia that is due to either an unbypassed artery or a coronary steal syndrome [3]. We present a case involving grafting of the saphenous to the left posterolateral coronary vein and a new technique to closure fistula with covered stent implantation into saphenous vein graft.

## 2. Case Report

**Figure 1:**
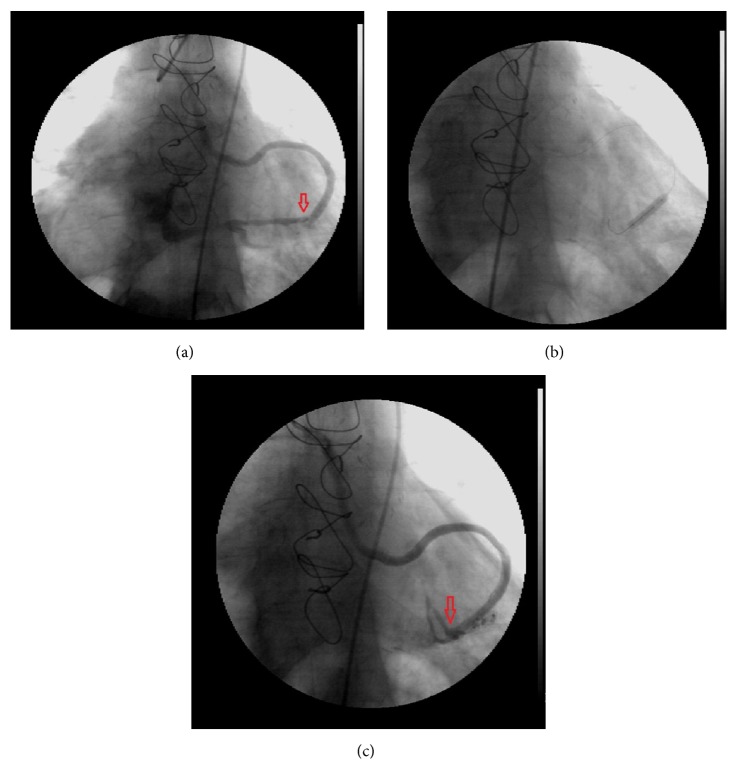
(a) As shown in the figure, the first sequential side to side ligation was inadvertently anastomosed to the left posterolateral coronary vein. (b) The covered stent was implanted at the fistula side. (c) The fistula was totally occluded and a TIMI 3 flow from saphenous vein graft to obtuse marginal artery was observed.

An 83-year-old man was admitted to the hospital with syncope and chest pain. His medical history included hypertension, diabetes mellitus, coronary artery bypass graft operation LIMA to LAD with sequential saphenous vein graft to first and second OM branches of circumflex artery 5 years ago, and coronary artery stent implantation to right coronary artery 2 months ago. On physical examination, blood pressure was 130/60 mmHg and heart rate was 60 beats/minute. A grade 2/6 mesocardiac continuous murmur was heard. Serum troponin I levels were normal. Twelve lead-ECG detected normal sinus rhythm and 0.4 seconds PR prolongation. Transthoracic echocardiography demonstrated biatrial enlargement, normal left ventricular systolic function, and grade 1 diastolic dysfunction. Because of the ongoing chest pain, a coronary angiography was performed. LAD artery was occluded after the first diagonal branch; LIMA to LAD was patent. There was a mild stenosis at the midportion of right coronary artery and a stent at the crux was patent. Circumflex artery was diffusely diseased. Aortosaphenous vein graft selective angiography revealed that the first sequential side to side ligation was inadvertently anastomosed to left posterolateral coronary vein with resultant flow into the coronary sinus and distal end to side sequential anastomosis to OM 2 coronary artery which was filling very weakly (Figure 1(a)). In order to close this iatrogenic coronary arteriovenous fistula and to supply saphenous vein graft flow to OM artery, we decided to implant a covered stent into the saphenous vein graft at the same session. A 4.0 × 9 mm covered stent (Abbott) was implanted at the fistula side. Control angiography showed incomplete occlusion of the fistula. Afterwards a second 4.0 × 13 mm covered stent (Abbott) was implanted inside the first stent (Figure 1(b)). At last, fistula was totally occluded and a TIMI 3 flow from saphenous vein graft to OM artery was observed (Figure 1(c)). On physical examination, cardiac murmur disappeared. Because of the extremely long PR prolongation and syncope, we implanted a DDD-R pacemaker. The patient was discharged with good recovery.

## 3. Discussion

Inadvertently, anastomosis of the coronary artery bypass grafts to coronary veins is seen rarely [4]. Only a total of 37 case reports have been recorded. The reasons of this complication may be anatomical distortion due to myocardial scarring, pericardial fibrosis, or previous coronary bypass operations. Also pericardial fat may disturb coronary exploration [5–7]. This iatrogenic coronary arteriovenous fistula may result in myocardial ischemia, infective endocarditis, severe systemic to pulmonary shunt, heart failure with high cardiac output, and fistula rupture [8]. Previous case reports revealed that this iatrogenic situation was treated with either redo coronary artery bypass grafting, balloon or coil embolization, stenting of the ungrafted artery, or covered stent implantation in the cardiac vein [9–11]. Spontaneously, closure of the asymptomatic fistula was documented in 2 cases [12]. To the best of our knowledge, for the first time in literature, we present treatment of iatrogenic coronary arteriovenous fistula with covered stent implantation to saphenous vein graft. It is easier and practical to implant ad hoc covered stent in the coronary artery compared to that in the cardiac vein during coronary angiography procedure. It is known that high restenosis rate is a disadvantage of covered stents. However, in this case, restenosis may consolidate the closure of fistula and probable myocardial ischemia due to the fact that restenosis can be managed with a repeat intervention although the myocardial perfusion area of this graft is not so significant. We suggest that this method has considerable advantages compared to redo bypass surgery because of its less invasive nature, and, in comparison with coil embolization, it does not have a risk of coil immigration.

## 4. Conclusion

Herein, we reported an aortocoronary arteriovenous fistula by deployment of intracoronary covered stent with a simple and safe method compared to other treatment options.
